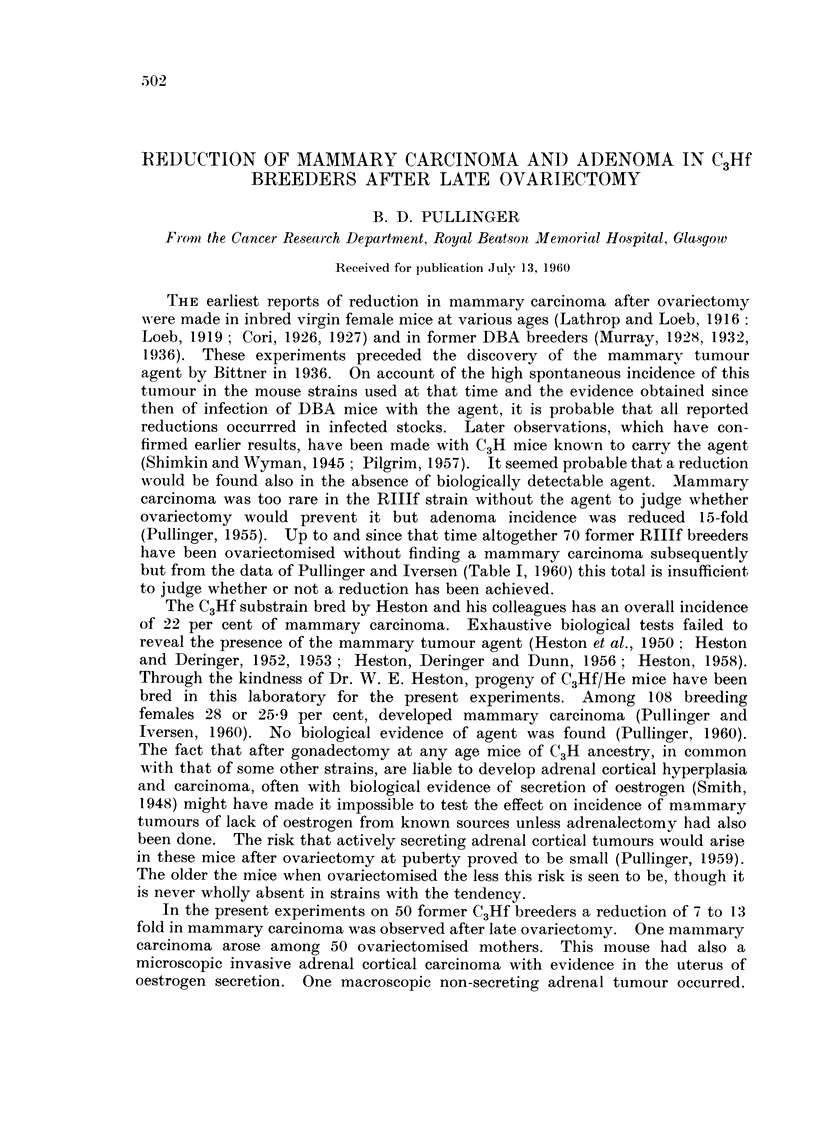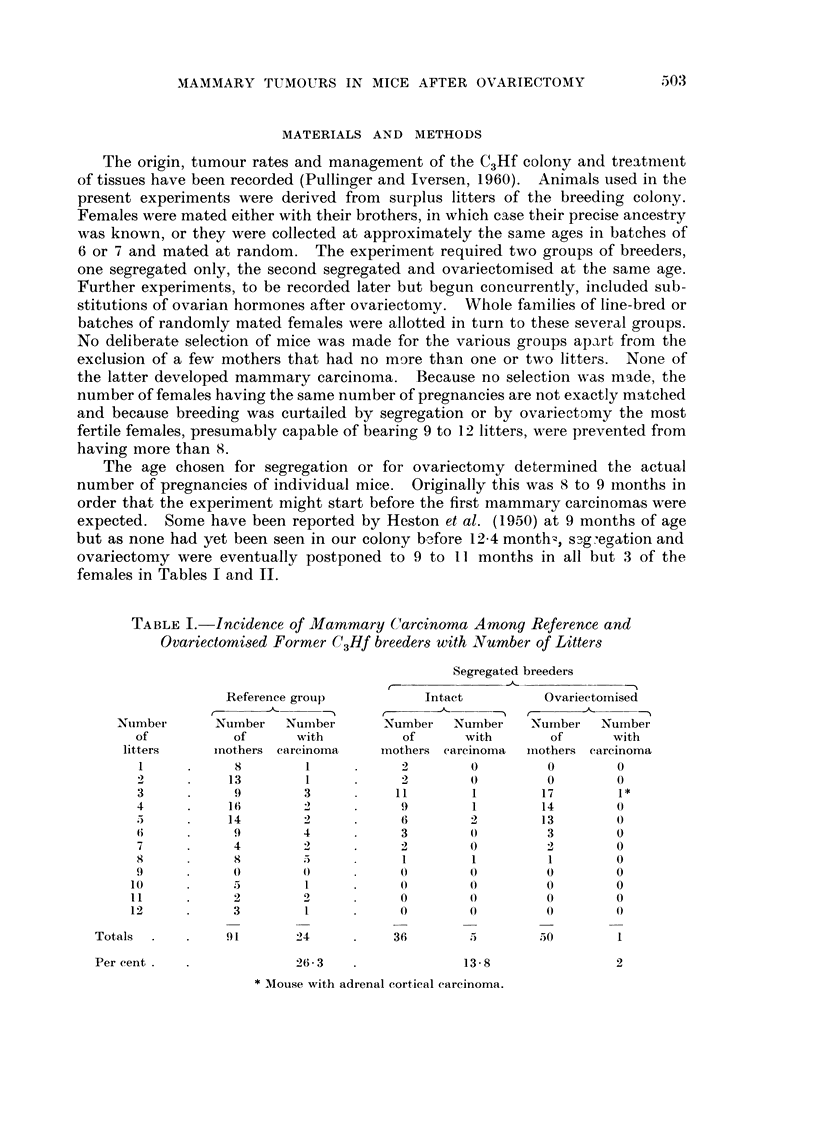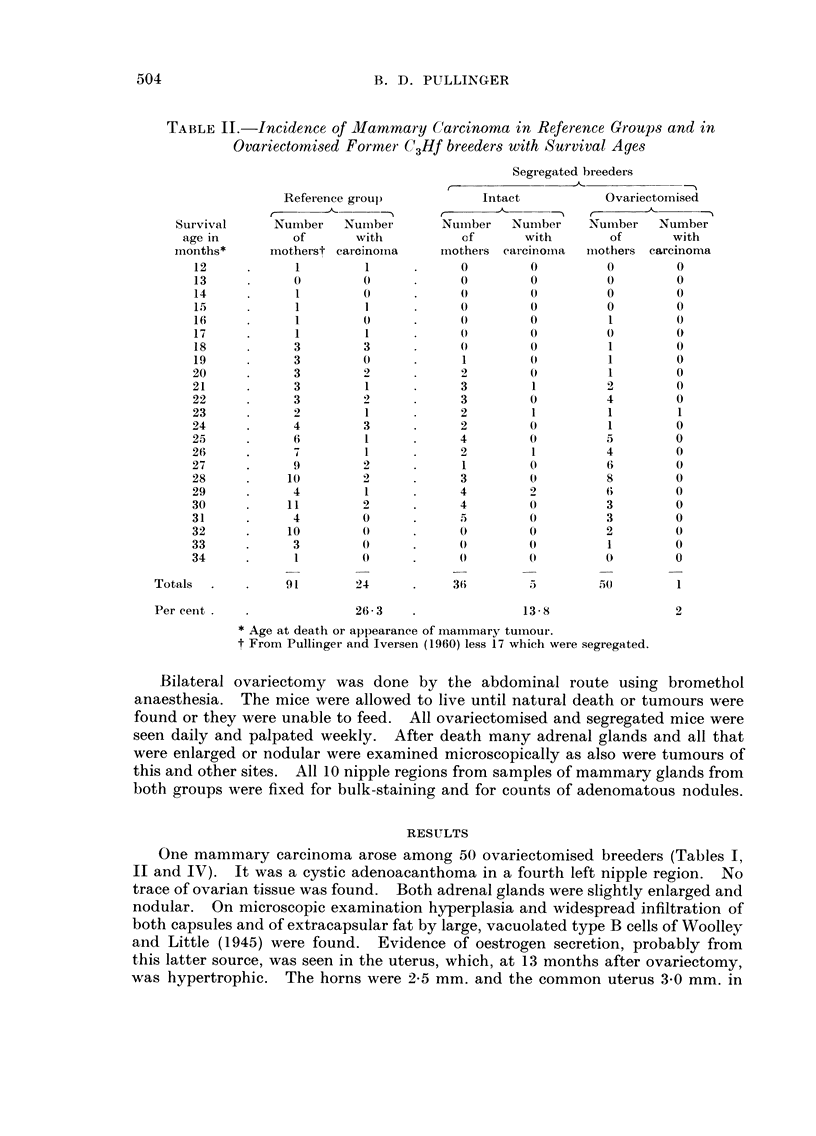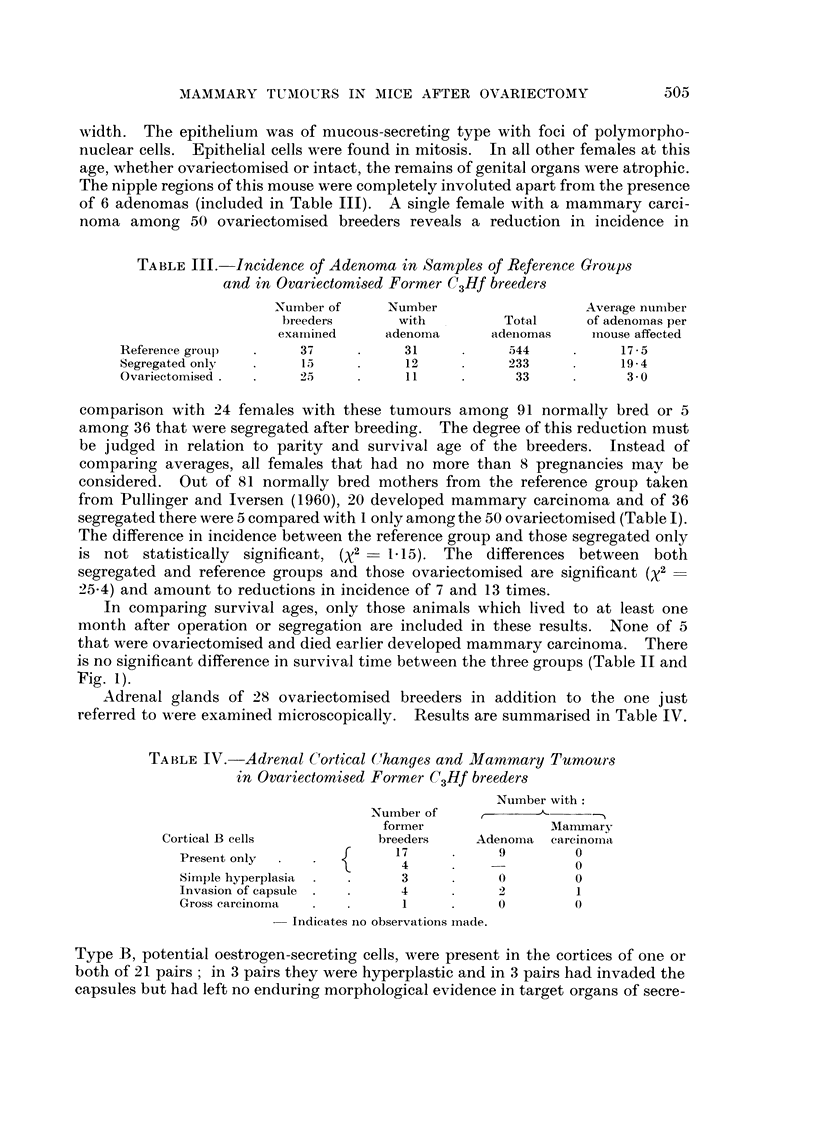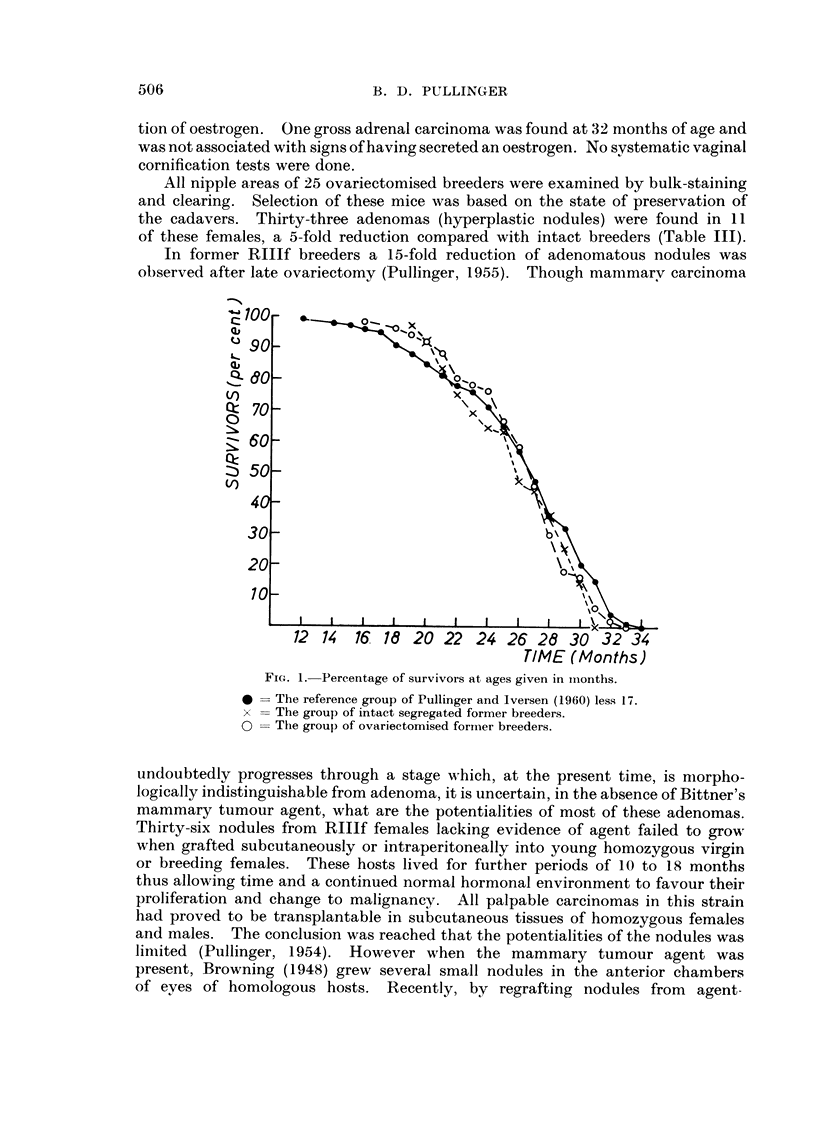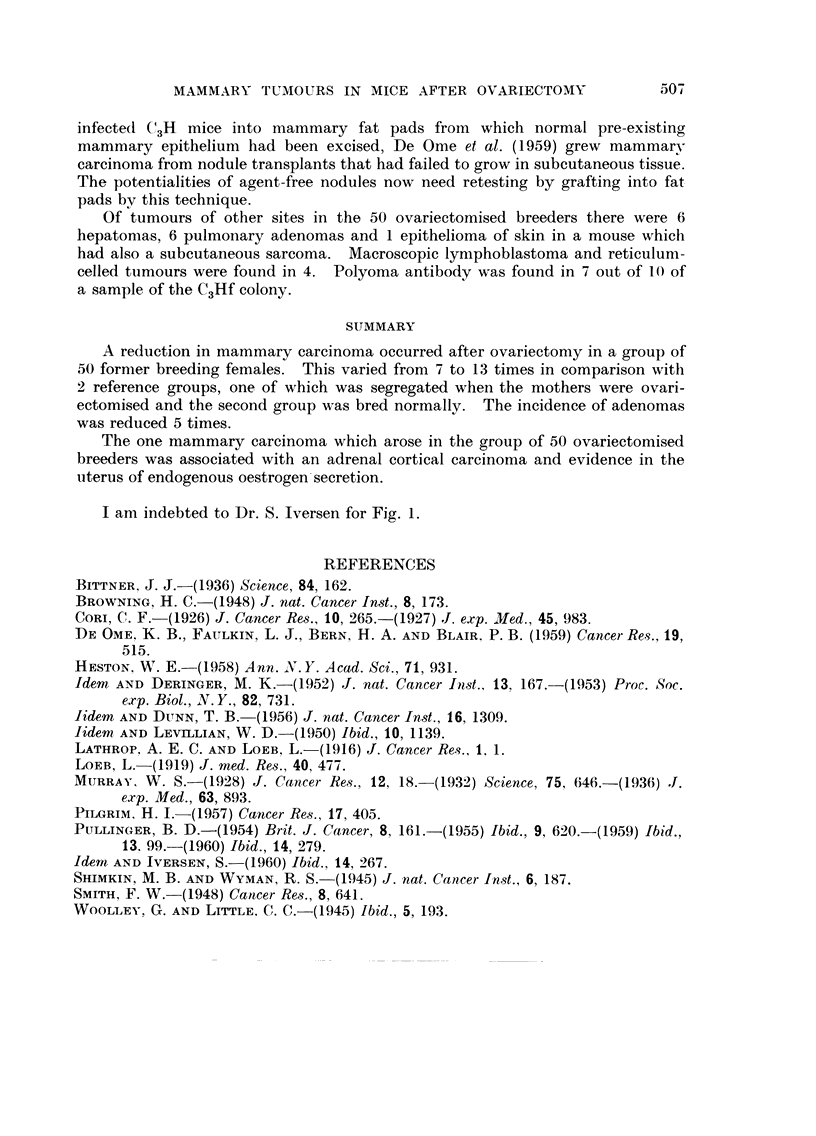# Reduction of Mammary Carcinoma and Adenoma in C_3_Hf Breeders after Late Ovariectomy

**DOI:** 10.1038/bjc.1960.55

**Published:** 1960-09

**Authors:** B. D. Pullinger


### REDUCTION OF MAMMARY CARCINOMA AND ADENOMA IN $C_3Hf$ BREEDERS AFTER LATE OVARIECTOMY

B. D. PULLINGER

*From the Cancer Research Department, Royal Beatson Memorial Hospital, Glasgow*

Received for publication July 13, 1960

THE earliest reports of reduction in mammary carcinoma after ovariectomy were made in inbred virgin female mice at various ages (Lathrop and Loeb, 1916 ; Loeb, 1919 ; Cori, 1926, 1927) and in former DBA breeders (Murray, 1928, 1932, 1936). These experiments preceded the discovery of the mammary tumour agent by Bittner in 1936. On account of the high spontaneous incidence of this tumour in the mouse strains used at that time and the evidence obtained since then of infection of DBA mice with the agent, it is probable that all reported reductions occurrred in infected stocks. Later observations, which have confirmed earlier results, have been made with $C_3H$ mice known to carry the agent (Shimkin and Wyman, 1945 ; Pilgrim, 1957). It seemed probable that a reduction would be found also in the absence of biologically detectable agent. Mammary carcinoma was too rare in the RIIIf strain without the agent to judge whether ovariectomy would prevent it but adenoma incidence was reduced 15-fold (Pullinger, 1955). Up to and since that time altogether 70 former RIIIf breeders have been ovariectomised without finding a mammary carcinoma subsequently but from the data of Pullinger and Iversen (Table I, 1960) this total is insufficient to judge whether or not a reduction has been achieved.

The $C_3Hf$ substrain bred by Heston and his colleagues has an overall incidence of 22 per cent of mammary carcinoma. Exhaustive biological tests failed to reveal the presence of the mammary tumour agent (Heston *et al.*, 1950 ; Heston and Deringer, 1952, 1953 ; Heston, Deringer and Dunn, 1956 ; Heston, 1958). Through the kindness of Dr. W. E. Heston, progeny of $C_3Hf$/He mice have been bred in this laboratory for the present experiments. Among 108 breeding females 28 or 25·9 per cent, developed mammary carcinoma (Pullinger and Iversen, 1960). No biological evidence of agent was found (Pullinger, 1960). The fact that after gonadectomy at any age mice of $C_3H$ ancestry, in common with that of some other strains, are liable to develop adrenal cortical hyperplasia and carcinoma, often with biological evidence of secretion of oestrogen (Smith, 1948) might have made it impossible to test the effect on incidence of mammary tumours of lack of oestrogen from known sources unless adrenalectomy had also been done. The risk that actively secreting adrenal cortical tumours would arise in these mice after ovariectomy at puberty proved to be small (Pullinger, 1959). The older the mice when ovariectomised the less this risk is seen to be, though it is never wholly absent in strains with the tendency.

In the present experiments on 50 former $C_3Hf$ breeders a reduction of 7 to 13 fold in mammary carcinoma was observed after late ovariectomy. One mammary carcinoma arose among 50 ovariectomised mothers. This mouse had also a microscopic invasive adrenal cortical carcinoma with evidence in the uterus of oestrogen secretion. One macroscopic non-secreting adrenal tumour occurred.

### MATERIALS AND METHODS

The origin, tumour rates and management of the $C_3Hf$ colony and treatment of tissues have been recorded (Pullinger and Iversen, 1960). Animals used in the present experiments were derived from surplus litters of the breeding colony. Females were mated either with their brothers, in which case their precise ancestry was known, or they were collected at approximately the same ages in batches of 6 or 7 and mated at random. The experiment required two groups of breeders, one segregated only, the second segregated and ovariectomised at the same age. Further experiments, to be recorded later but begun concurrently, included substitutions of ovarian hormones after ovariectomy. Whole families of line-bred or batches of randomly mated females were allotted in turn to these several groups. No deliberate selection of mice was made for the various groups apart from the exclusion of a few mothers that had no more than one or two litters. None of the latter developed mammary carcinoma. Because no selection was made, the number of females having the same number of pregnancies are not exactly matched and because breeding was curtailed by segregation or by ovariectomy the most fertile females, presumably capable of bearing 9 to 12 litters, were prevented from having more than 8.

The age chosen for segregation or for ovariectomy determined the actual number of pregnancies of individual mice. Originally this was 8 to 9 months in order that the experiment might start before the first mammary carcinomas were expected. Some have been reported by Heston *et al.* (1950) at 9 months of age but as none had yet been seen in our colony before 12·4 months, segregation and ovariectomy were eventually postponed to 9 to 11 months in all but 3 of the females in Tables I and II.

TABLE I.—*Incidence of Mammary Carcinoma Among Reference and Ovariectomised Former $C_3Hf$ breeders with Number of Litters*

| | Reference group | | Segregated breeders: Intact | | Segregated breeders: Ovariectomised | |
|---|---|---|---|---|---|---|
| Number of litters | Number of mothers | Number with carcinoma | Number of mothers | Number with carcinoma | Number of mothers | Number with carcinoma |
| 1 | 8 | 1 | 2 | 0 | 0 | 0 |
| 2 | 13 | 1 | 2 | 0 | 0 | 0 |
| 3 | 9 | 3 | 11 | 1 | 17 | 1* |
| 4 | 16 | 2 | 9 | 1 | 14 | 0 |
| 5 | 14 | 2 | 6 | 2 | 13 | 0 |
| 6 | 9 | 4 | 3 | 0 | 3 | 0 |
| 7 | 4 | 2 | 2 | 0 | 2 | 0 |
| 8 | 8 | 5 | 1 | 1 | 1 | 0 |
| 9 | 0 | 0 | 0 | 0 | 0 | 0 |
| 10 | 5 | 1 | 0 | 0 | 0 | 0 |
| 11 | 2 | 2 | 0 | 0 | 0 | 0 |
| 12 | 3 | 1 | 0 | 0 | 0 | 0 |
| Totals | 91 | 24 | 36 | 5 | 50 | 1 |
| Per cent | | 26·3 | | 13·8 | | 2 |

* Mouse with adrenal cortical carcinoma.

TABLE II.—*Incidence of Mammary Carcinoma in Reference Groups and in Ovariectomised Former $C_3Hf$ breeders with Survival Ages*

| | Reference group | | Segregated breeders: Intact | | Segregated breeders: Ovariectomised | |
|---|---|---|---|---|---|---|
| Survival age in months* | Number of mothers† | Number with carcinoma | Number of mothers | Number with carcinoma | Number of mothers | Number with carcinoma |
| 12 | 1 | 1 | 0 | 0 | 0 | 0 |
| 13 | 0 | 0 | 0 | 0 | 0 | 0 |
| 14 | 1 | 0 | 0 | 0 | 0 | 0 |
| 15 | 1 | 1 | 0 | 0 | 0 | 0 |
| 16 | 1 | 0 | 0 | 0 | 1 | 0 |
| 17 | 1 | 1 | 0 | 0 | 0 | 0 |
| 18 | 3 | 3 | 0 | 0 | 1 | 0 |
| 19 | 3 | 0 | 1 | 0 | 1 | 0 |
| 20 | 3 | 2 | 2 | 0 | 1 | 0 |
| 21 | 3 | 1 | 3 | 1 | 2 | 0 |
| 22 | 3 | 2 | 3 | 0 | 4 | 0 |
| 23 | 2 | 1 | 2 | 1 | 1 | 1 |
| 24 | 4 | 3 | 2 | 0 | 1 | 0 |
| 25 | 6 | 1 | 4 | 0 | 5 | 0 |
| 26 | 7 | 1 | 2 | 1 | 4 | 0 |
| 27 | 9 | 2 | 1 | 0 | 6 | 0 |
| 28 | 10 | 2 | 3 | 0 | 8 | 0 |
| 29 | 4 | 1 | 4 | 2 | 6 | 0 |
| 30 | 11 | 2 | 4 | 0 | 3 | 0 |
| 31 | 4 | 0 | 5 | 0 | 3 | 0 |
| 32 | 10 | 0 | 0 | 0 | 2 | 0 |
| 33 | 3 | 0 | 0 | 0 | 1 | 0 |
| 34 | 1 | 0 | 0 | 0 | 0 | 0 |
| Totals | 91 | 24 | 36 | 5 | 50 | 1 |
| Per cent | | 26·3 | | 13·8 | | 2 |

* Age at death or appearance of mammary tumour.
† From Pullinger and Iversen (1960) less 17 which were segregated.

Bilateral ovariectomy was done by the abdominal route using bromethol anaesthesia. The mice were allowed to live until natural death or tumours were found or they were unable to feed. All ovariectomised and segregated mice were seen daily and palpated weekly. After death many adrenal glands and all that were enlarged or nodular were examined microscopically as also were tumours of this and other sites. All 10 nipple regions from samples of mammary glands from both groups were fixed for bulk-staining and for counts of adenomatous nodules.

### RESULTS

One mammary carcinoma arose among 50 ovariectomised breeders (Tables I, II and IV). It was a cystic adenoacanthoma in a fourth left nipple region. No trace of ovarian tissue was found. Both adrenal glands were slightly enlarged and nodular. On microscopic examination hyperplasia and widespread infiltration of both capsules and of extracapsular fat by large, vacuolated type B cells of Woolley and Little (1945) were found. Evidence of oestrogen secretion, probably from this latter source, was seen in the uterus, which, at 13 months after ovariectomy, was hypertrophic. The horns were 2·5 mm. and the common uterus 3·0 mm. in width. The epithelium was of mucous-secreting type with foci of polymorphonuclear cells. Epithelial cells were found in mitosis. In all other females at this age, whether ovariectomised or intact, the remains of genital organs were atrophic. The nipple regions of this mouse were completely involuted apart from the presence of 6 adenomas (included in Table III). A single female with a mammary carcinoma among 50 ovariectomised breeders reveals a reduction in incidence in

TABLE III.—*Incidence of Adenoma in Samples of Reference Groups and in Ovariectomised Former $C_3Hf$ breeders*

| | Number of breeders examined | Number with adenoma | Total adenomas | Average number of adenomas per mouse affected |
|---|---|---|---|---|
| Reference group | 37 | 31 | 544 | 17·5 |
| Segregated only | 15 | 12 | 233 | 19·4 |
| Ovariectomised | 25 | 11 | 33 | 3·0 |

comparison with 24 females with these tumours among 91 normally bred or 5 among 36 that were segregated after breeding. The degree of this reduction must be judged in relation to parity and survival age of the breeders. Instead of comparing averages, all females that had no more than 8 pregnancies may be considered. Out of 81 normally bred mothers from the reference group taken from Pullinger and Iversen (1960), 20 developed mammary carcinoma and of 36 segregated there were 5 compared with 1 only among the 50 ovariectomised (Table I). The difference in incidence between the reference group and those segregated only is not statistically significant, ($\chi^2 = 1{\cdot}15$). The differences between both segregated and reference groups and those ovariectomised are significant ($\chi^2 = 25{\cdot}4$) and amount to reductions in incidence of 7 and 13 times.

In comparing survival ages, only those animals which lived to at least one month after operation or segregation are included in these results. None of 5 that were ovariectomised and died earlier developed mammary carcinoma. There is no significant difference in survival time between the three groups (Table II and Fig. 1).

Adrenal glands of 28 ovariectomised breeders in addition to the one just referred to were examined microscopically. Results are summarised in Table IV.

TABLE IV.—*Adrenal Cortical Changes and Mammary Tumours in Ovariectomised Former $C_3Hf$ breeders*

| Cortical B cells | Number of former breeders | Number with: Adenoma | Mammary carcinoma |
|---|---|---|---|
| Present only | 17 | 9 | 0 |
| | 4 | — | 0 |
| Simple hyperplasia | 3 | 0 | 0 |
| Invasion of capsule | 4 | 2 | 1 |
| Gross carcinoma | 1 | 0 | 0 |

— Indicates no observations made.

Type B, potential oestrogen-secreting cells, were present in the cortices of one or both of 21 pairs ; in 3 pairs they were hyperplastic and in 3 pairs had invaded the capsules but had left no enduring morphological evidence in target organs of secretion of oestrogen. One gross adrenal carcinoma was found at 32 months of age and was not associated with signs of having secreted an oestrogen. No systematic vaginal cornification tests were done.

All nipple areas of 25 ovariectomised breeders were examined by bulk-staining and clearing. Selection of these mice was based on the state of preservation of the cadavers. Thirty-three adenomas (hyperplastic nodules) were found in 11 of these females, a 5-fold reduction compared with intact breeders (Table III).

In former RIIIf breeders a 15-fold reduction of adenomatous nodules was observed after late ovariectomy (Pullinger, 1955). Though mammary carcinoma

FIG. 1.—Percentage of survivors at ages given in months.

● = The reference group of Pullinger and Iversen (1960) less 17.
× = The group of intact segregated former breeders.
○ = The group of ovariectomised former breeders.

undoubtedly progresses through a stage which, at the present time, is morphologically indistinguishable from adenoma, it is uncertain, in the absence of Bittner's mammary tumour agent, what are the potentialities of most of these adenomas. Thirty-six nodules from RIIIf females lacking evidence of agent failed to grow when grafted subcutaneously or intraperitoneally into young homozygous virgin or breeding females. These hosts lived for further periods of 10 to 18 months thus allowing time and a continued normal hormonal environment to favour their proliferation and change to malignancy. All palpable carcinomas in this strain had proved to be transplantable in subcutaneous tissues of homozygous females and males. The conclusion was reached that the potentialities of the nodules was limited (Pullinger, 1954). However when the mammary tumour agent was present, Browning (1948) grew several small nodules in the anterior chambers of eyes of homologous hosts. Recently, by regrafting nodules from agentinfected $C_3H$ mice into mammary fat pads from which normal pre-existing mammary epithelium had been excised, De Ome *et al.* (1959) grew mammary carcinoma from nodule transplants that had failed to grow in subcutaneous tissue. The potentialities of agent-free nodules now need retesting by grafting into fat pads by this technique.

Of tumours of other sites in the 50 ovariectomised breeders there were 6 hepatomas, 6 pulmonary adenomas and 1 epithelioma of skin in a mouse which had also a subcutaneous sarcoma. Macroscopic lymphoblastoma and reticulum-celled tumours were found in 4. Polyoma antibody was found in 7 out of 10 of a sample of the $C_3Hf$ colony.

### SUMMARY

A reduction in mammary carcinoma occurred after ovariectomy in a group of 50 former breeding females. This varied from 7 to 13 times in comparison with 2 reference groups, one of which was segregated when the mothers were ovariectomised and the second group was bred normally. The incidence of adenomas was reduced 5 times.

The one mammary carcinoma which arose in the group of 50 ovariectomised breeders was associated with an adrenal cortical carcinoma and evidence in the uterus of endogenous oestrogen secretion.

I am indebted to Dr. S. Iversen for Fig. 1.

### REFERENCES

BITTNER, J. J.—(1936) *Science*, **84**, 162.
BROWNING, H. C.—(1948) *J. nat. Cancer Inst.*, **8**, 173.
CORI, C. F.—(1926) *J. Cancer Res.*, **10**, 265.—(1927) *J. exp. Med.*, **45**, 983.
DE OME, K. B., FAULKIN, L. J., BERN, H. A. AND BLAIR, P. B. (1959) *Cancer Res.*, **19**, 515.
HESTON, W. E.—(1958) *Ann. N.Y. Acad. Sci.*, **71**, 931.
*Idem* AND DERINGER, M. K.—(1952) *J. nat. Cancer Inst.*, **13**, 167.—(1953) *Proc. Soc. exp. Biol., N.Y.*, **82**, 731.
*Iidem* AND DUNN, T. B.—(1956) *J. nat. Cancer Inst.*, **16**, 1309.
*Iidem* AND LEVILLIAN, W. D.—(1950) *Ibid.*, **10**, 1139.
LATHROP, A. E. C. AND LOEB, L.—(1916) *J. Cancer Res.*, **1**, 1.
LOEB, L.—(1919) *J. med. Res.*, **40**, 477.
MURRAY, W. S.—(1928) *J. Cancer Res.*, **12**, 18.—(1932) *Science*, **75**, 646.—(1936) *J. exp. Med.*, **63**, 893.
PILGRIM, H. I.—(1957) *Cancer Res.*, **17**, 405.
PULLINGER, B. D.—(1954) *Brit. J. Cancer*, **8**, 161.—(1955) *Ibid.*, **9**, 620.—(1959) *Ibid.*, **13**, 99.—(1960) *Ibid.*, **14**, 279.
*Idem* AND IVERSEN, S.—(1960) *Ibid.*, **14**, 267.
SHIMKIN, M. B. AND WYMAN, R. S.—(1945) *J. nat. Cancer Inst.*, **6**, 187.
SMITH, F. W.—(1948) *Cancer Res.*, **8**, 641.
WOOLLEY, G. AND LITTLE, C. C.—(1945) *Ibid.*, **5**, 193.